# Excessive Oxidative Stress Contributes to Increased Acute ER Stress Kidney Injury in Aged Mice

**DOI:** 10.1155/2019/2746521

**Published:** 2019-01-28

**Authors:** Xiaoyan Liu, Ruihua Zhang, Lianghu Huang, Zihan Zheng, Helen Vlassara, Gary Striker, Xiaoyan Zhang, Youfei Guan, Feng Zheng

**Affiliations:** ^1^Department of Nephrology, The Second Hospital and Center for Renal Diseases, Advanced Institute for Medical Sciences, Dalian Medical University, Dalian, Liaoning 116044, China; ^2^Department of Urology, Dongfang Hospital, Fujian Medical University, Fuzhou, Fujian 350009, China; ^3^Department of Geriatrics, Mount Sinai School of Medicine, New York City, NY 10029, USA

## Abstract

The aged kidney is susceptible to acute injury due presumably to its decreased ability to handle additional challenges, such as endoplasmic reticulum (ER) stress. This was tested by giving tunicamycin, an ER stress inducer, to either old or young mice. Injection of high dose caused renal failure in old mice, not in young mice. Moreover, injection of low dose resulted in severe renal damage in old mice, confirming the increased susceptibility of aged kidney to ER stress. There existed an abnormality in ER stress response kinetics in aged kidney, characterized by a loss of XBP-1 splicing and decreased PERK-eIF2*α* phosphorylation at late time point. The presence of excessive oxidative stress in aged kidney may play a role since high levels of oxidation increased ER stress-induced cell death and decreased IRE1 levels and XBP-1 splicing. Importantly, treatment with antioxidants protected old mice from kidney injury and normalized IRE1 and XBP-1 responses. Furthermore, older mice (6 months old) transgenic with antioxidative stress AGER1 were protected from ER stress-induced kidney injury. In conclusion, the decreased ability to handle ER stress, partly due to the presence of excessive oxidative stress, may contribute to increased susceptibility of the aging kidney to acute injury.

## 1. Introduction

Older individuals are more susceptible to renal failure caused by drug toxicity and ischemic injury [1, 2]. Several factors, such as the existence of mild to moderate renal function decline, abnormalities in drug turnover, and dysregulation of the vasculature, have been implicated in the pathogenesis of aging-related susceptibility [2–5]. Since the kidney naturally also faces an increased oxidant load with declining antioxidant activity over the course of aging [4–6], we postulated that the above factors in combination may lead to a decreased capability in aging kidney to defend against acute stressors.

Endoplasmic reticulum (ER) stress is a common form of cell stress. The accumulation of unmodified or unfolded proteins in the ER induces a stress response called the unfolded protein response (UPR) [7–9]. The UPR serves to reduce protein synthesis, accelerates the folding machinery, and increases the degradation of unfolded proteins to prevent buildup of unfolded proteins. Thus far, three separate pathways have been identified that regulate the UPR, including pancreatic ER kinase (PERK), activating transcription factor 6 (ATF6), and the inositol requiring enzyme 1 (IRE1) and X-box binding protein 1 (XBP-1) pathways [10–12]. These three pathways are activated in an attempt to reduce the stress. Failure to suppress ER stress may result in increased generation of reactive oxygen species (ROS), inflammation, and cell death. Increased acute stress has been shown to contribute to various diseases including diabetes mellitus, neurodegenerative diseases, cardiac disease, and atherosclerosis [9, 13, 14].

ER stress in the kidney has recently been shown to be an underlying cause of acute drug-induced nephrotoxicity [15–19]. Moreover, hypoxia and ischemia have been shown to generate ER stress [20–22]. We postulate that the aged kidney has a decreased capability to cope with acute increases in ER stress due to the presence of factors such as oxidative stress [4, 5, 23]. This loss in coping capability may contribute to the increased susceptibility of the aged kidney to drug and ischemic injury.

## 2. Methods

### 2.1. Mice

18–22-month-old C57B6 female mice, purchased from the National Institute on Aging, National Institutes of Health, Bethesda, MD, were housed in a specific pathogen-free facility with free access to regular diet and water. Since aged C57B6 mice may develop malignancies, we examined all mice and excluded those with tumors from the study. 3–6-month-old C57B6 female mice were purchased from Jackson Laboratories (Bar Harbor, ME). AGER1 transgenic mice were generated, bred to C57B6 background, and characterized as previously described [24]. 6-month-old female mice were used for the study. All experiments and animal care procedures were performed according to the Guide to Animal Use and Care of the Dalian Medical University, and every effort was made to minimize suffering. This study was approved by the Institutional Animal Care Use Committee of Dalian Medical University.

### 2.2. Tunicamycin Induced Acute ER Stress

To select a dose of tunicamycin that would induce renal damage but not mortality, 3–6-month-old female C57B6 mice were injected intraperitoneally with the following doses of tunicamycin: 0.6, 0.8, 1.0, 1.2, and 1.4 *μ*g/g/body weight in 0.9% NaCl (*n* = 2/dose). Mice were followed for 4 days. Renal histology and renal function indicators of blood urea nitrogen (BUN) levels were examined. Severe proximal tubular injury and increased BUN levels were found in young mice at a dose above 1.2 *μ*g/g. Since a 0.8 *μ*g/g dose generated moderate renal proximal tubular injury, without increasing serum BUN levels, this dose (designated at high dose) was chosen to determine if the response to ER stress injury differed between old (*n* = 22) and young mice (*n* = 22). Mice were sacrificed at 24 hours (*n* = 8/age), 48 hours (*n* = 6/age), and 72 hours (*n* = 8/age) after injection. After examining tunicamycin doses from 0.2 to 0.6 *μ*g/g to find a dose that would induce significant kidney damage in old mice but not in young mice, the dose of 0.2 *μ*g/g was selected. Both old (*n* = 18) and young mice (*n* = 18) were injected with this dose of tunicamycin. Mice were sacrificed 24, 48, and 72 hours after injection for blood and tissue collection. Additionally, 6-month-old AGER1 transgenic and wild-type mice (*n* = 14/group) were given 0.8 *μ*g/g of tunicamycin and sacrificed at 24 hours (*n* = 7/group) and 72 hours (*n* = 7/group) after injection.

### 2.3. N-Acetylcysteine (NAC) and Butylated Hydroxyanisole (BHA) Treatment

18–22 months and 3–6 months C57B6 mice (*n* = 12/age group) received NAC (150 *μ*g/g/body weight) intraperitoneally 24 hours before the injection of tunicamycin (0.8 *μ*g/g). Mice were sacrificed 48 hours or 72 hours after tunicamycin challenge. For BHA preventive treatment, BHA (0.7%) was added to regular mouse chow of old and young mice for 7 days before tunicamycin injection (0.8 *μ*g/g). Mice were sacrificed 48 hours (*n* = 6/age group) or 72 hours (*n* = 6/age group) after tunicamycin challenge.

### 2.4. Analysis of Renal Function and Tunicamycin Blood Level

Blood samples were collected from animals at sacrifice. BUN was measured using a QuantiChrom assay kit (QuantiChrom™ Urea Assay Kit, BioAssay Systems, Hayward, Calif.) following the manufacturer's instructions. Serum creatinine was measured by high-performance liquid chromatography (HLPC) using creatinine (Sigma, Munich, Germany) as standard. Plasma tunicamycin levels were determined by HPLC using tunicamycin (Sigma, Munich Germany) as standard.

### 2.5. Histology and Morphometry

Kidneys were flushed with phosphate-buffered saline and then fixed in 4% paraformaldehyde for 48 hours. After fixation, tissues were washed and transferred to phosphate-buffered saline. Tissues were embedded in glycol methacrylate or low-melting point paraffin, and sections were cut at a thickness of 4 *μ*m and stained with periodic acid Schiff (PAS) [25]. For electron microscopy, tissues were fixed for 1 hour in 1.0% osmium tetroxide, prestained in 1.25% uranyl acetate for 1 hour, dehydrated through a series of graded alcohol solutions, and embedded in EPON epoxy resin. The severity of histological lesions in tunicamycin-treated old and young mice was determined using a Meta Imaging software (Molecular Devices, Downingtown, PA, USA). The injured proximal tubules in the cortical area were digitized under a microscope connected with a Sony 3CCD color video camera. The ratio of the damaged to normal tubular area was measured [26].

### 2.6. Apoptotic Cell Staining

Paraffin sections were processed for staining as described [27, 28]. Briefly, tissues were digested with 20 *μ*g/ml of proteinase K for 2.5 minutes and then reacted with terminal deoxynucleotidyl transferase (TdT) for 1 hour. Positive reactions were revealed by peroxidase-conjugated antidigoxigenin and DAB. Nuclei were stained with hematoxylin. The number of apoptotic cells was counted under 400x magnification. At least 10 random fields in each section were examined.

### 2.7. Western Blots

Renal cortices were collected under microscope guidance. Tissue proteins were extracted using a lysis buffer containing proteinase inhibitors [27]. Equal amounts of protein were loaded in each lane of sodium dodecyl sulfate polyacrylamide gels. After electrophoresis, proteins were transferred to nitrocellulose membrane and blotted with antibody against GRP78 (1 : 5000, Affinity BioReagents (ABR), Golden, CO), GRP-94 (1 : 5000, Affinity BioReagents (ABR), Golden, CO), phosphorylated eIF2*α* (1 : 1000, Stressgen, Ann Arbor, MI), C/EBP homologous protein (CHOP, 1 : 1000, Alexis Biochemicals, San Diego, CA), nuclear poly ADP-ribose polymerase (PARP, 1 : 1000, Cell Signaling Technology, Danvers, MA), or caspase 12 (1 : 1000, Cell Signaling Technology, Danvers, MA) [26]. Membranes probed for phosphorylated eIF2*α* were stripped to reblot with antibody against total eIF2*α* (1 : 5000, Bethyl Laboratories, Montgomery, TX). Finally, all membranes were stripped to reprobe for *β*-actin as an internal control. The blotting for each molecule was repeated at least twice. For the measurement of phosphorylated PERK, 100 *μ*g of tissue protein from the kidneys of young and old mice was immunoprecipitated with a rabbit anti-PERK antibody (Santa Cruz Biotechnology Inc., Santa Cruz, CA). The precipitates were recovered with protein A/G agarose (Pierce Biotechnologies, Rockfold, IL). After denaturation, the immunoprecipitated protein was examined by Western blots for phosphorylated PERK with a specific antibody (SC32577-R, 1 : 200, Santa Cruz Biotechnology Inc., Santa Cruz, CA).

#### 2.7.1. Real-Time PCR

Total RNA was isolated from the renal cortex using a PureYield RNA Midiprep kit (Promega, Madison, WI). The preparation was free of DNA contamination. 500 ng of total RNA from each sample was reverse transcripted and amplified using SYBR® Premix Ex Taq™ II reagent kit and ABI Prism 7700 sequence detection system (PerkinElmer Applied Biosystems, Foster City, CA) as previously described [27]. The GRP78, GRP94, oxygen-regulated protein 150 (ORP150), ER degradation-enhancing *α*-mannosidase I-like protein (EDEM1), CHOP, and IRE1 mRNA levels were determined. The primers were from previous reports [26, 29]: GRP78, forward, 5′-TACTCGGGCCAAATTTGAAG, reverse, 5′-CATGGTAGAGCGGAACAGGT; GRP94, 5′-TGAAGGAGAAGCAGGACAAAA, reverse, 5′-AGTCGCTCAACAAAGGGAGA; ORP150 forward, 5′-GAAGCCAACCGGCTTAAAAC, reverse, 5′-CCGAGTTACTTTGGCCTTGA; EDEM1 forward, 5′-TGTGAAAGCCCTCTGGAACT, reverse, 5′-AATGGCCTGTCTGGATGTTC; CHOP forward, 5′-TATCTCATCCCCAGGAAACG, reverse, 5′-GGACGCAGGGTCAAGAGTAG. IRE1 forward, 5′-TGAAACACCCCTTCTTCTGG, reverse, 5′-CAGGGGGACAGTGATGTTCT. The mRNA levels were corrected by the levels of *β*-actin mRNA. Triplicates for each sample were done to increase accuracy of the measurement. However, since both previous reports [26, 29] had not described a 5-log dilution for primers and we had not specifically optimized PCR conditions, the levels of gene expression measured here were only semiquantitative in nature although the △△Ct values were calculated after real-time PCR.

### 2.8. Spliced XBP-1

Total RNA was extracted from the renal cortex and reversed transcribed as described [23]. When we screened for dosages of tunicamycin for experiments, we noticed that X-box binding protein 1 (XBP-1) splicing occurred at 48 hours after tunicamycin treatment. Thus, this time point was chosen for examining XBP-1. The presence of the spliced XBP-1 mRNA levels was measured by standard PCR using the following primers: forward, 5′-TTACGGGAGAAAACTCACGGC; reverse, 5′-GGGTCCAACTTGTCCAGAATGC. 40 cycles of PCR were performed with the annealing temperature of 58°C. GAPDH mRNA levels were measured at the same sample using the previously described primers [26, 27].

### 2.9. Analysis of Oxidative Stress

Lipid peroxidation in the kidney was determined by a thiobarbituric acid-reactive substance assay that measures the formation of malondialdehyde (MDA) (Cayman Chemical, Ann Arbor, MI) [25]. The quantity of protein carbonyls in the kidney was measured by first reacting kidney extracts with dinitrophenylhydrazine (DNP). Protein-bound DNP was then detected by a biotinylated anti-DNP antibody followed by streptavidin-linked horseradish peroxidase. Absorbances were related to a standard (Chemicon International, Temecula, CA). To determine the levels of glutathione, kidney extracts were processed and deproteinized. A chemical reaction kit (Cayman Chemical, Ann Arbor, MI) was used to measure glutathione. Both the levels of reduced and total glutathione were determined. Kidney AGE levels were determined by ELISA and corrected for protein levels as previously described [30].

### 2.10. Oxidative Stress and Unfolded Protein Response in Proximal Tubular Cells

A proximal tubular cell line obtained from mice transgenic for SV40 T antigen was grown in DMEM containing 10% FBS [31]. To determine if oxidative stress could directly affect UPR, proximal tubular cells were treated with H_2_O_2_ (0.5–3 mM) in the presence or absence of NAC (15 mM, adding 1 hour before H_2_O_2_) for 6–24 hours. IRE1 mRNA and protein and XBP-1 splicing and protein were determined.

### 2.11. Proximal Tubule Isolation and Tunicamycin-Induced Cell Death

Three mice from young or old mice were sacrificed, and renal cortical proximal tubules (PT) were isolated by a standard method [25, 32]. Briefly, renal cortexes were freed from the medulla under a dissecting microscope. Tissues were then cut into 1-2 mm^3^ pieces and digested with collagenase. Proximal tubules were obtained from gradient Percoll centrifugation. Proximal tubules were suspended in DMEM containing 2% FBS, allocated to a 24-well plate, and incubated with increasing concentration of tunicamycin (0.5–5 *μ*g/ml). Both media and tubular segments were collected 24 hours after tunicamycin treatment to determine LDH activity.

### 2.12. Statistical Analysis

Data were expressed as mean ± SD. ANOVA or the two-tailed unpaired *t*-test was used to evaluate differences between the means. For comparison with more than two subgroups, the nonparametric Kruskal-Wallis ANOVA followed by Dunnett's test was performed. Significance was defined as *p* < 0.05.

## 3. Results

### 3.1. Tunicamycin-Induced Acute ER Stress Is more Severe in Older Mice

**Figure 1:**
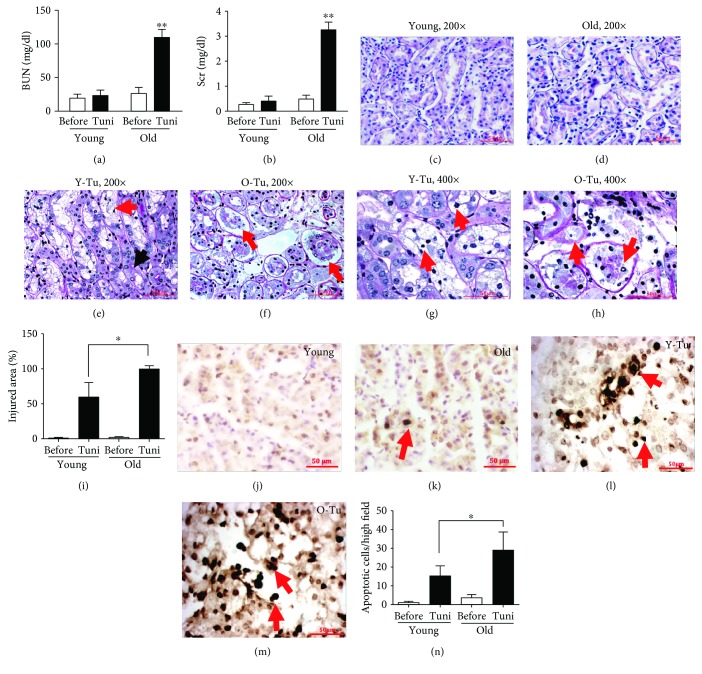
Differences in kidney function and renal lesions between old and young mice after high dose of tunicamycin injury (0.8 *μ*g/g BW, *n* = 8/age group). (a, b) Increased BUN and Scr levels in old mice after high dose of tunicamycin injection: serum samples were obtained from 18–22-month- or 3–6-month-old mice prior to and after 72 hours of high dose of tunicamycin injection. (c, d) Representative kidney sections from (c) young and (d) old mice without receiving tunicamycin seem normal. (e, g) Extensive tubular vacuolation was present in young mice at 72 hours of high dose tunicamycin injury (PAS, (e) 200x, and (g) 400x). The lesions were localized in proximal tubules (red arrow) while the descending tubules extended from the injured proximal tubule were relatively normal as indicated by a (e) black arrow. (g) Nuclear pyknosis (condensation) and fragmentation were widely present (arrows). (f) Large vacuoles were less common in the kidneys of tunicamycin-treated old mice (200x). (f) However, the cellular damage was more severe with detachment of the whole segment of proximal tubular cells from the basement membrane in old mice (arrows). (h) Higher power magnification (400x) showed that cells in the injured tubules in aging kidney contained many small or fine vacuoles. Nuclear damages were also prominent. (i) Morphometry analysis revealed that tubular damage occurred nearly in all proximal tubules in old mice after high dose of tunicamycin injection. More apoptotic cell death in aging kidney after high dose of tunicamycin injury: apoptotic TUNEL staining was performed in kidney sections obtained from (l) young or (m) old mice at 72 hours of high-dose tunicamycin injection. Kidneys without tunicamycin treatment for (j) young and (k) old mice were as controls (400x). Nuclei with brown or black staining (DAB) were apoptotic cells (arrows). (n) More apoptotic cell counts in the kidneys from tunicamycin-treated old mice. ^∗^ *p* < 0.05 vs. young mice. ^∗∗^ *p* < 0.01 vs. the levels before tunicamycin injection. Data was expressed as mean ± SD. Scale bar = 50 *μ*m.

Tunicamycin, an inhibitor of protein N-glycosylation, which has been used extensively to induce ER stress *in vitro* and *in vivo* [33–35], was given to mice (0.8 *μ*g/g). A sharp decrease in renal function as reflected by elevated BUN and Scr levels was observed in older mice (Figures 1(a) and 1(b)). Results from several of our experiments including this study on young AGER1 transgenic wild-type controls indicated that the BUN and Scr levels remained unchanged in most of young mice treated with 0.8 *μ*g/g of tunicamycin (data not shown), demonstrating that younger mice can better tolerate acute ER stress. Detailed pathological and histological investigations further revealed the age-related differences in acute ER stress response. The renal tubulointerstitial area appears normal in both young and old mice before the tunicamycin challenge (Figures 1(c) and 1(d)). Cortical enlargement caused by swelling and vacuolation of proximal tubular cells was observed in young mice after tunicamycin treatment, while other tubular segments remained largely intact (Figure 1(e)). Cell death under light microscopy, as evidenced by chromatin condensation, pyknosis, and nuclear fragmentation (Figure 1(g)), could be seen in the main lesions in tunicamycin-treated young mice, which occurred in about 60% of proximal tubules (Figure 1(i)). In old mice treated with tunicamycin, tubular injury was very severe and occurred in 94% of the area (Figure 1(i)). Damages presented as the detachment of the whole proximal tubular epithelium from the basement membrane (Figures 1(f) and 1(h)). TUNEL staining showed a 2-fold increase in apoptosis in tubular cells in old mice (Figures 1(j)–1(n)). Taken together, these results demonstrate that older mice are much more susceptible to ER stress than younger mice.

### 3.2. Low-Dose Tunicamycin-Induced Acute ER Stress Leading to Renal Lesions in Old Mice

Since tunicamycin at a dose of 0.8 *μ*g/g also caused kidney injury in young mice, we then sought to reduce the dosage of tunicamycin to rule out the possibility that results observed were solely because of the extreme degree of ER stress received. At a dose of 0.2 *μ*g/g, young mice developed very trivial tubular lesions (affecting less than 5% proximal tubules) (Supplementary Figures 1(a) and (c)). However, the kidney lesions in old mice were widespread, with 60% of the areas showing extensive proximal tubular damage consisting of vacuolation, cell swelling, and cell death (Supplementary Figures 1(b) and (c)). TUNEL staining showed more apoptotic cells in the kidneys of old mice (14.3 ± 6.7 per high-power field) than in the kidneys of young mice (3.7 ± 2.8 per high-power field, ^∗∗^ *p* < 0.01) (Supplementary Figures 1(d)–(f)).

### 3.3. ER Stress Induces Proximal Tubular Injury in Old Mice

The above results clearly showed that old mice were more susceptible to ER stress-induced kidney injury *in vivo*. One of the primary reasons for increased drug nephrotoxicity experienced by older individuals is their decreased ability to eliminate drugs, leading to higher blood/tissue concentration and/or prolonged exposure. To find out if this is the case for tunicamycin, we measured its concentration in plasma from young or old mice at 1, 2, and 24 hours after 0.8 *μ*g/g of tunicamycin intraperitoneal injection. Plasma tunicamycin was detectable at 1 hour after drug injection, and there was no difference in the levels between old and young mice (Supplementary Figure 2), which argues against delayed drug excretion as a major cause of increased tunicamycin nephrotoxicity in old mice. Our current and previous studies have shown that proximal tubules are major sites for ER stress-induced kidney injury [26]. Thus, we isolated proximal tubules from either old or young mice to test directly their response to acute ER stress injury. Tunicamycin caused a dose-dependent increase in proximal tubular cell death (Supplementary Figure 3). When exposed to the same dose of tunicamycin, more cell death occurred in proximal tubules from old mice (Supplementary Figure 3), suggesting that proximal tubules from the old are more susceptible to injury.

On the organelle level, electron microscopy examination showed that changes of proximal tubular cells in young mice were mild (Supplementary Figure 4(a)), while there were extensive vacuolar changes in old mice (Supplementary Figure 4(b)). Higher definition further showed that abnormal ER and mitochondria were widely present in old mice (Supplementary Figures 4(c) and (d)). Mitochondria were condensed with many electron-dense bodies absent of their cristae (Supplementary Figure 4(c)). Additionally, the vacuoles in proximal tubular cells observed under light microscopy and low magnification of electron microscopy seemed to be dilated rough ER, as evidenced by the presence of ribosomes on the surface of the vacuoles (Supplementary Figure 4(d)). Overall, it is clear that proximal tubular cells in older mice are much more susceptible to acute ER stress, while those of younger mice are able to tolerate it.

### 3.4. Prolonged UPR Responses in the Kidneys of Old Mice after Low Dose of Tunicamycin Injury (0.2 *μ*g/g)

Chemical and genetic manipulations of UPR have been shown to directly affect the outcome of acute ER stress-induced injury, and aging has been linked with changes in UPR mechanisms [35–40]. Based on these reports, we next examined the expression of UPR-related genes before and after tunicamycin treatment that might explain the age-linked difference in ER stress. Prior to tunicamycin treatment, the mRNA baseline levels of ER chaperones GRP78 and GRP94 were about 50% and 60% lower, respectively, in the kidneys of old mice than in the kidneys of young mice (*n* = 6/age group, Supplementary Figure 5). Additionally, the mRNA levels of protective ORP150 and IRE1 were also lower in the kidneys from old mice (Supplementary Figure 5). Surprisingly however, the baseline levels of GRP78 and GRP94 proteins were similar between the kidneys of old and young mice (Supplementary Figure 7(b)), despite a sizeable difference in mRNA expression. The cause of the discrepancies between mRNA and protein levels is not clear. One of the reasons may be that our real-time PCR conditions especially the primers have not been optimized by 5-log dilutions and may affect the accuracy of mRNA quantitation. Another reason may be the unparallel expression between mRNAs and proteins of GRP78 and GRP94 in the kidney. To test this, we dissected out proximal and distal tubules for PCR and performed immunostaining of proteins in different nephron segments. We found by both regular and real-time PCR that GRP78 and GRP94 mRNA levels were higher in proximal tubules than in distal tubules (data not shown), while the proteins were higher in distal tubules than in proximal tubules (Supplementary Figure 6), suggesting that posttranscriptional modification or transport mechanism was in play in different nephron segments.

Low dose (0.2 *μ*g/g) of tunicamycin was sufficient to induce ER stress in both the kidneys of young and old mice as indicated by the appearance of spliced XBP-1 at 48 hours (Supplementary Figure 7(a)). However, the levels of GRP78, GRP94, OPR150, EDEM1, and CHOP mRNAs were higher in the kidneys of old mice after 72 hours (Supplementary Figure 7(a)). GRP78 and GRP94 protein levels were also elevated in old kidneys. Moreover, CHOP and cleaved caspase 12 were present in old kidneys (Supplementary Figures 7(b) and (c)), consistent with *in vivo* observations of higher tubular apoptosis and severe renal injury in old mice 72 hours after acute ER stress (Supplementary Figure 1).

### 3.5. Dysregulated UPR Responses in the Kidneys of Old Mice after High Dose of Tunicamycin Injury (0.8 *μ*g/g)

**Figure 2:**
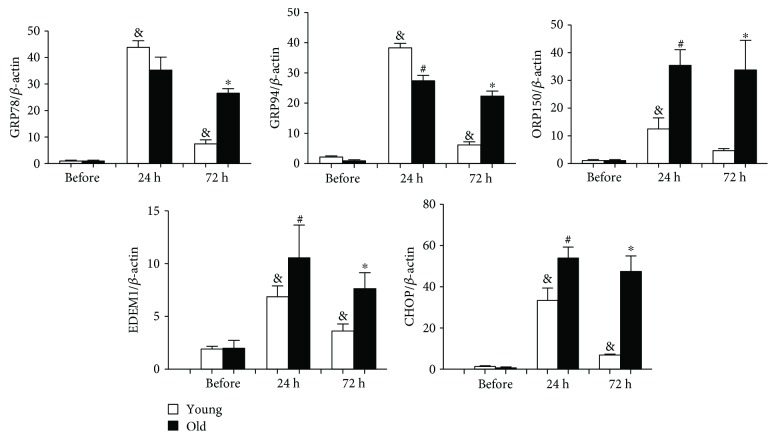
Differences in mRNA levels of UPR-related genes between the kidneys of old and young mice after high dose of tunicamycin injury: renal cortex RNA was obtained from young and old mice at baseline, 24 hours, and 72 hours after high dose of tunicamycin (0.8 *μ*g/g) injection. The levels of GRP78, GRP94, ORP150, EDEM1, and CHOP mRNAs were measured by real-time PCR, and data was expressed as the ratio after dividing with *β*-actin mRNA levels at the same sample. ^&^ *p* < 0.05 vs. young mice at baseline. ^#^ *p* < 0.05 vs. young mice at 24 hours. ^∗^ *p* < 0.05 vs. young mice at 72 hours.

**Figure 3:**
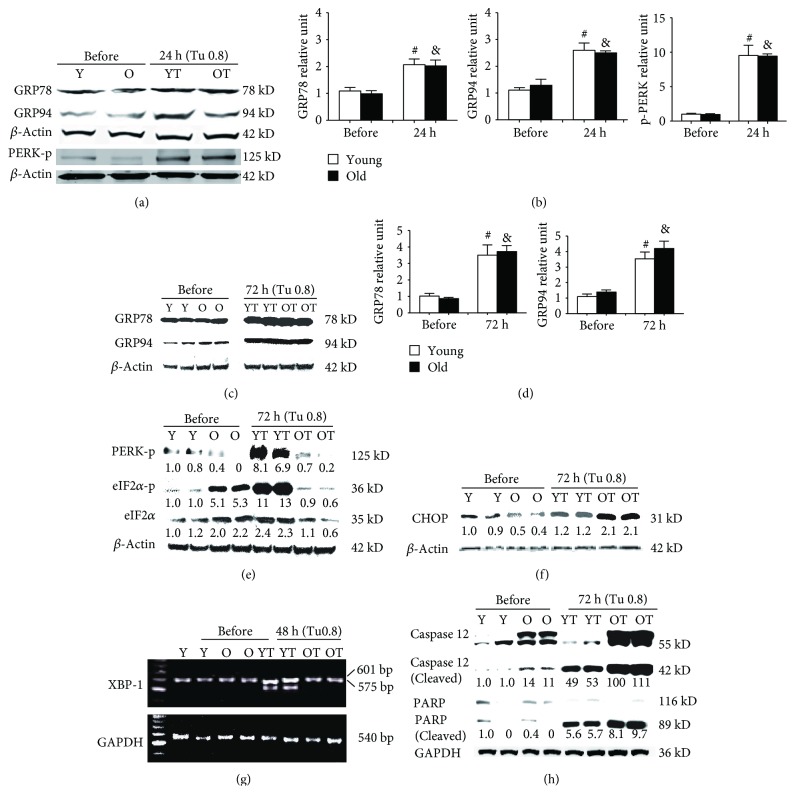
Abnormalities in UPR in the kidneys of old mice: renal cortex RNA was obtained from young and old mice at baseline, 24 hours, 48 hours, and 72 hours after high dose of tunicamycin (0.8 *μ*g/g) injection. The levels of GRP78, GRP94, phosphorylated PERK (PERK-p), phosphorylated eIF2*α* (eIF2*α*-p), total eIF2*α*, CHOP, caspase 12, and PARP were measured in eight animals from each group by Western blots. XBP-1 and GAPDH mRNA expression in kidneys at baseline and 48 hours after tunicamycin injection was determined by RT-PCR (*n* = 6). Results from two representative animals of baseline and tunicamycin-treated young and old mice were shown. The intensity of each blot band was quantitated using a densitometer. Data from the untreated kidneys of young mice was arbitrarily defined as 1 after correcting with the intensity of the individual *β*-actin band of the same sample. Lanes Y (baseline) and YT (tunicamycin treated) were samples from young mice. Lanes O (baseline) and OT (tunicamycin treated) were samples from old mice. Both GRP78 and GRP94 were significantly increased in the kidneys of both young and old mice at 24 (a, b) and 72 hours (c, d) after tunicamycin treatment. No differences were found between young and old mice. At baseline, phosphorylated PERK levels were low (a, e). The levels were significantly elevated in both old and young mice 24 hours after high dose of tunicamycin injection (a). Phosphorylated PERK remained high in young mice but was largely lost in old mice at 72 hours (e). Subsequently, at 72 hours, phosphorylated eIF2*α* was increased in young mice while it was decreased in old mice (e). High-dose tunicamycin treatment was associated with more increased kidney CHOP protein levels in old mice (f). XBP-1 mRNA splicing occurred only in the kidneys of young mice after treatment (g). Cleaved caspase 12 levels were increased in old mice at baseline and were further increased after tunicamycin treatment (h). The increase in cleaved PARP was also more robust in old mice (h). ^#^ *p* < 0.05 vs. young mice at baseline. ^&^ *p* < 0.05 vs. old mice at baseline.

To further explore the differences in UPR response in the kidneys of old and young mice, we also examined mice treated with a high dose of tunicamycin (0.8 *μ*g/g). The mRNAs GRP78 and GRP94 were increased in both old and young mice 24 hours after tunicamycin injection (Figure 2). Similarly, ORP150, EDEM1, and CHOP mRNAs were upregulated in both old and young mice at 24 hours but the increases were more pronounced in the old (Figure 2). 72 hours later, the levels of GRP78, GRP94, OPR150, EDEM1, and CHOP mRNAs had significantly reduced in young mice while they remained high in old mice (Figure 2). However, at the protein levels, GRP78 and GRP94 proteins were similarly increased in the kidneys from old and young mice at both 24- and 72-hour time points (Figures 3(a)–3(d)). Thus, the unparallel expression between mRNA and protein levels of GRP78 and GRP94 in the kidneys still existed after being exposed to high dose of tunicamycin. Nevertheless, XBP-1 splicing was found missing in old mice receiving high dose of tunicamycin (Figure 3(g)). Additionally, the increase in phosphorylated PERK, although occurring in old mice at 24 hours (Figure 3(a)), was largely lost at 72 hours (Figure 3(e)). Subsequently, phosphorylated eIF2*α* was also largely missed in old mice at 72 hours (Figure 3(e)). These were associated with highly elevated proapoptotic CHOP, cleaved caspase 12, and cleaved PARP proteins in old mice (Figures 3(f) and 3(h)). In contrast, XBP-1 splicing was clearly present (Figure 3(g)) and phosphorylated PERK were increased at both 24- and 72-hour time points in young mice (Figures 3(a) and 3(e)), which were accompanied by increased phosphorylated eIF2*α* (Figure 3(e)).

### 3.6. Inhibition of Oxidative Stress Largely Protected Old Mice from High-Dose Tunicamycin-Induced Renal Injury

**Figure 4:**
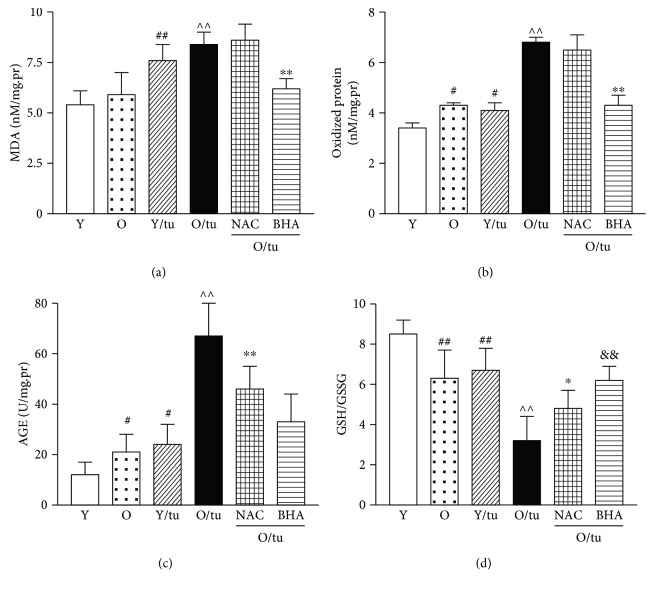
ER stress-induced severe oxidative stress in the kidneys of old mice is prevented by antioxidants. Renal tissue was obtained from young and old mice at baseline, 72 hours after tunicamycin (0.8 *μ*g/g) injection and old mice pretreatment with BHA for 7 days or NAC 24 hours before tunicamycin injection. ER stress caused elevation in MDA, oxidized protein, and AGEs and decreased reducing glutathione (GSH)/glutathione disulfide (GSSG). (a) Levels of MDA, (b) levels of oxidized protein, (c) levels of AGEs, and (d) the ratio of GSH and GSSG. Y: young mice; O: old mice; Y/tuni: young mice with tunicamycin injury; O/tuni: old mice with tunicamycin injury: NAC; old mice treated with NAC; BHA: old mice treated with BHA. ^#^ *p* < 0.05 and ^##^ *p* < 0.01 vs. young mice at baseline; ^∧∧^ *p* < 0.01 vs. old mice at baseline; ^∗^ *p* < 0.05 and ^∗∗^ *p* < 0.01 vs. old mice that received tunicamycin; and ^&&^ *p* < 0.01 vs. old mice treated with NAC.

Oxidative stress has been shown elevated in aging kidney [11, 12]. Since ER stress causes oxidative stress and oxidative stress in turn contributes to ER stress-induced cell death, we postulated that excessive oxidative stress might play a role in severe ER stress-induced kidney injury in old mice. We first assessed the state of oxidative stress in kidneys from old and young mice prior to and after tunicamycin treatment. Although baseline levels of malondialdehyde (MDA), one of the parameters for lipid peroxidation, were comparable between old and young mice (Figure 4(a)), the levels of oxidized proteins and advanced glycation end products (AGEs) were significantly elevated in the kidneys of old mice (Figures 4(b) and 4(c)). Additionally, the ratio of reduced to oxidized glutathione (GSH/GSSG) was decreased in the kidneys of old mice, confirming the presence of increased oxidative stress in the kidneys of old mice (Figure 4(d)). After high dose of tunicamycin injection, the levels of MDA in the kidneys were increased comparably by about 44% in both old and young mice (Figure 4(a)). However, the levels of oxidized protein and AGEs were more elevated in old mice following treatment (Figures 4(b) and 4(c)). At the same time, the GSH/GSSG ratio for older mice was halved by ER stress, while younger mice exhibited a modest decline (Figure 4(d)). To determine if high levels of oxidative stress directly contribute to increased ER stress-induced kidney injury in old mice, old mice were given antioxidants butylated hydroxyanisole (BHA) or N-acetyl cysteine (NAC) before tunicamycin. Interestingly, both BHA treatment and NAC treatment helped to preserve reduced glutathione levels in the kidneys (Figure 4(d)). In addition, BHA treatment seemed to have a better antioxidant effect, because it also significantly downregulated MDA (Figure 4(a)). Consequently, the elevation of oxidized protein levels after tunicamycin injury was completely blocked by BHA (Figure 4(b)). The levels of AGEs were also significantly decreased by antioxidants, with much higher reduction in BHA-treated than in NAC-treated old mice (Figure 4(c)).

**Figure 5:**
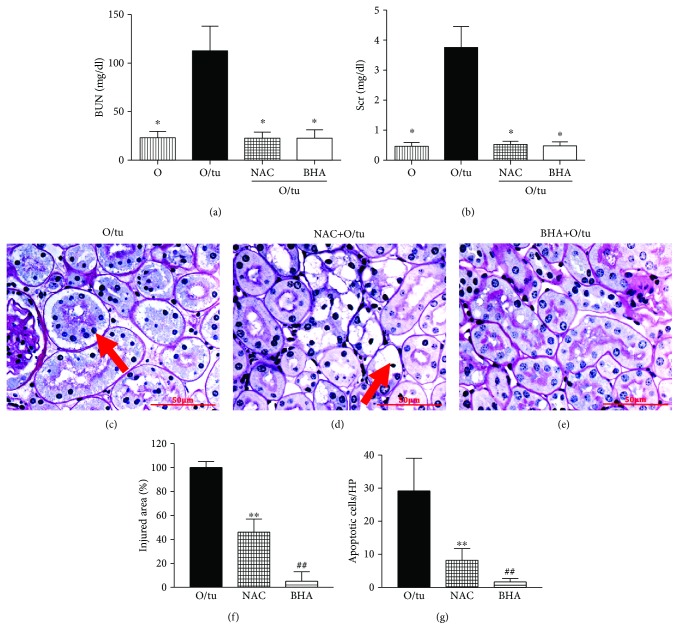
Prevention of ER stress renal injury in old mice by antioxidants. NAC or BHA treatment blocked high dose of tunicamycin-induced elevation (0.8 *μ*g/g) of BUN (a) and Scr (b). Histologically, while severe renal injury characterized by sloughing of tubular cells (arrow) was frequently seen in old mice that received tunicamycin alone (c), NAC treatment resulted in a partial protection against tunicamycin-induced renal injury in old mice (d). There is no tubular cell sloughing, but vacuolation and nuclear damage (arrow) were seen in NAC-treated mice (d). BHA treatment gave a nearly complete protection, and renal histology was essentially normal in this group (e). Morphometry quantitation of the injured area in the renal cortex revealed a significant reduction in ER stress renal injury by antioxidants, and the effect was especially prominent by BHA (f). Counting the number of TUNEL-positive cells in kidney sections showed that BHA and NAC decreased tunicamycin-induced apoptotic cell death (g). ^∗∗^ *p* < 0.01 vs. old mice that received tunicamycin alone (O/tu) and ^##^ *p* < 0.01 vs. NAC-treated mice. Scale bar = 50 *μ*m.

Importantly, both NAC and BHA prevented renal function decline induced by severe ER stress. Unlike untreated old mice that had significantly elevated BUN and Scr after tunicamycin injury (Figures 5(a) and 5(b)), the BUN and Scr levels were normal in mice receiving NAC or BHA. Renal histological examination results were consistent with the functional data, demonstrating substantial improvements upon treatment with either NAC or BHA. The severe lesions featuring proximal tubular cell sloughing were not seen in NAC- or BHA-treated old mice (Figures 5(d) and 5(e)). The main lesions in NAC-treated old mice (Figure 5(d)) were the vacuoles in proximal tubular cells instead of severe damages characterized by detachment of tubular cells seen in controls (Figure 5(c)). Only mild lesions were present in BHA-treated old mice (Figure 5(e)). Quantitative measurement of the area of injury showed that BHA treatment was able to reduce the coverage from 98% to 5%, while NAC treatment was able to halve it (Figure 5(f)). The number of apoptotic cells remained directly correlated with the severity of renal lesions (Figures 5(f) and 5(g)). Old mice treated only with tunicamycin exhibited extensive apoptotic cell death, which was largely prevented by antioxidants, with BHA being more effective than NAC (Figure 5(g)). Collectively, these results demonstrate that BHA treatment is highly effective for reducing the impact of severe ER stress on proximal tubular cells in aged mice.

### 3.7. Inhibition of Oxidative Stress Largely Corrected the Altered UPR in Aging Kidney

**Figure 6:**
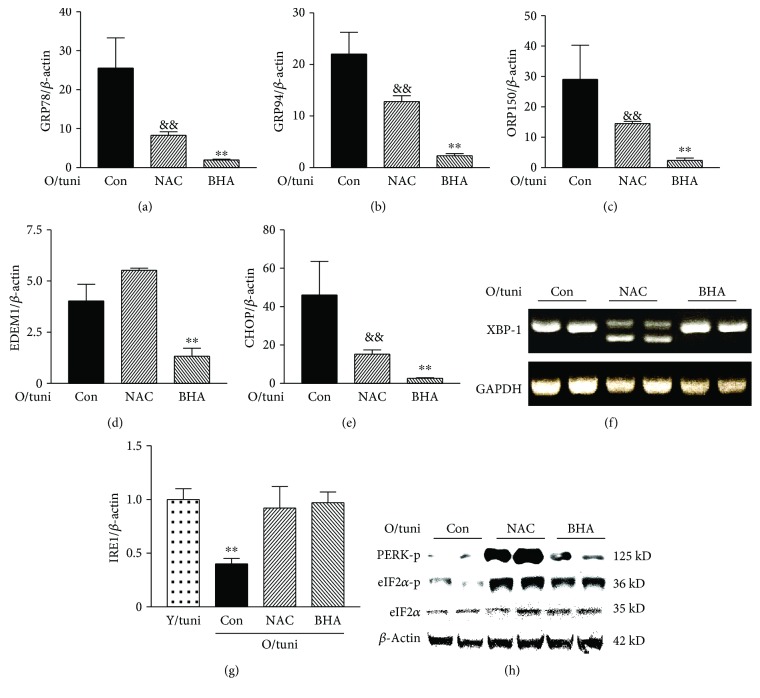
Antioxidant treatment corrects UPR dysregulation in old mice. RNA was collected from the kidneys of controls that received tunicamycin alone (0.8 *μ*g/g) or treated with NAC or BHA before tunicamycin injection (*n* = 6/group). mRNA levels of GRP78 (a), GRP94 (b), OPR150 (c), EDEM1 (d), and CHOP (e) in the kidneys at 72 hours after tunicamycin injection were determined by real-time PCR and corrected for *β*-actin mRNA levels. O/tuni: old mice challenged with high dose of tunicamycin. ^&&^ *p* < 0.01 vs. old mice treated with tunicamycin alone (Con). ^∗∗^ *p* < 0.01 vs. mice pretreated with NAC. XBP-1 spicing (f). Regular PCR was performed in the kidneys from tunicamycin alone (Con) and NAC- and BHA-treated mice at 48 hours after tunicamycin injection (*n* = 6/group). Representative gels from two mice of each group were shown. The loss of XBP-1 splicing in tunicamycin alone (Con) mice reappears in NAC-treated mice. IRE1 mRNA levels (g). ^∗∗^ *p* < 0.01 vs. young mice treated with high dose of tunicamycin or old mice pretreated with NAC or BHA. The levels of phosphorylated PERK, phosphorylated eIF2*α*, and total eIF2*α* in the kidneys from control and NAC- and BHA-treated old mice (h). Renal protein was obtained from these mice 72 hours after tunicamycin injection (*n* = 6/group). Representative gel shows two samples from each group. The levels of phosphorylated PERK and eIF2*α* were highest in the NAC-treated group. Phosphorylated eIF2*α* was also visibly higher in the BHA group than in the control group.

Since antioxidant treatment significantly improved the outcome of ER stress renal injury in old mice, we then examined if antioxidant treatment also corrected dysregulated UPR dynamics. Real-time PCR results showed that mRNA levels of GRP78, GRP94, EDEM, and CHOP were significantly lower in old mice that received BHA or NAC treatment (*p* < 0.01, Figures 6(a)–6(e)). Importantly, NAC treatment led to the appearance of XBP-1 splicing (Figure 6(f)), a loss in untreated old mice after higher levels of acute ER stress kidney injury. However, no XBP-1 splicing was observed in BHA-treated mice (Figure 6(f)), one of the reasons may be that BHA treatment nearly completely protected the kidneys of old mice from acute ER stress injury. Both NAC treatment and BHA treatment prevented the decrease in IRE1 mRNA levels (Figure 6(g)). Additionally, NAC treatment and BHA treatment both restored the PERK-eIF2*α* pathway response to high dose of tunicamycin at the 72-hour time point (Figure 6(h)), providing the aged kidneys with another UPR pathway to relieve stress. Taken together, these data suggest that antioxidant treatment is highly effective to restore the kinetics of UPR pathways in response to severe ER stress in old mice.

### 3.8. Oxidants Decreased IRE1 and XBP-1 Splicing in Proximal Tubular Cells

Since ER stress-induced kidney injury occurred predominantly in proximal tubules and the injury could be prevented by antioxidant prophylaxis, we next asked if oxidative stress causes UPR dysregulation in proximal tubular cells. We tested this in a proximal tubular cell line with known overactive XBP-1 splicing. Cells were exposed to H_2_O_2_ in the presence or absence of NAC. As shown in Supplementary Figure 8, NAC blocked the suppression of IRE1 and XBP-1 on both mRNA and protein levels by H_2_O_2_. These results suggest that oxidative stress may have a direct effect on UPR regulation in proximal tubular cells.

### 3.9. Mice Transgenic for AGER1 Were Resistant to Tunicamycin-Induced ER Stress Kidney Injury

An aging kidney has an elevated oxidative stress due to decreased antioxidants/anticarbonyls and excessive oxidants/carbonyls. Since the above results show a clear role played by oxidative stress in driving acute ER stress renal injury, we then sought to determine whether overexpression of AGER1, one of the antioxidants/anticarbonyls that is decreased in aging kidney while the oxidants/carbonyls are more prevalent [30], was protective against acute ER stress kidney injury. Mice transgenic for AGER1 have been shown to be resistant to acute arterial wire injury [24]. AGER1 transgenic mice, which have a nearly 5-fold increase in AGER1 expression in the kidney [24], and wild-type mice were treated with tunicamycin (0.8 *μ*g/g). As shown above, renal injury characterized by extensive vacuolation and tubular cell death was widely present in wild-type mice (Supplementary Figure 9(a)) while AGER1 transgenic mice had an obvious reduction in injury lesions (Supplementary Figure 9(b)). The injured area was reduced by 50% in the kidneys of transgenic mice (Supplementary Figure 9(c)), demonstrating a significant protection from tunicamycin-induced acute ER stress kidney injury in AGER1 transgenic mice.

## 4. Discussion

It is well-known that the aging kidney is more susceptible to acute kidney injury and it develops more severe postinjury outcome due to the preexisting structural and functional deterioration [1, 41–43]. We show here that old mice, but not young mice, developed severe renal failure after high dose of tunicamycin injection. Even low dosage of tunicamycin (reduced as to cause minimal or no kidney damage in young mice) was sufficient to induce extensive injury and apoptosis in the kidneys of old mice. Since ER stress is commonly associated with acute kidney injury caused by infection, drug toxicity, and ischemia [16, 18, 19, 32, 44], the inability to handle ER stress may be another mechanism for increased susceptibility to and severity of acute injury in aging kidney.

There were noticeable differences in magnitude and temporality of GRP78, GRP94, and ORP150 expression between the kidneys of old and young mice at baseline and after tunicamycin treatment although there were inconsistencies between the levels of mRNAs and those of proteins in GRP78 and GRP94 due partly to posttranscription modifications. Nevertheless, based on the more severe kidney injury seen in old mice, we speculate that there may be a difference in UPR between the kidneys of old and young mice. Indeed, we found a dysregulated UPR dynamic in old mice: while UPR at 24 hours after high dose of tunicamycin treatment was similar between the kidneys of old and young mice, XBP-1 splicing was lost at 48 hours and phosphorylated PERK and eIF2*α* were not elevated at 72 hours in old mice. PERK and eIF2*α* phosphorylation along with XBP-1 splicing are two key steps in ER stress regulation. Although inhibition of IRE-1 has been shown to reduce apoptosis [36] and activation of PERK-eIF2*α* may promote apoptosis by inducing CHOP [36], these may not be a general phenomenon since both IRE1-XBP-1 signaling and PERK-eIF2*α* phosphorylation pathways have also been shown to be critical in maintaining cell survival during ER stress [36, 45]. Additionally, since high dose of tunicamycin caused extensive renal damage at 72 hours in the kidneys of old mice, the results of missing PERK and eIF2*α* phosphorylation and XBP-1 at a relative late time point likely reflected the failure of dynamic ER stress responses. The causes of this failure are not clear. Oxidative stress is one of the key factors in aging, along with a number of human pathological conditions, and has been shown to play an important role in ER stress-induced cell death [46–48]. Unsurprisingly, the kidneys from old mice had higher basal levels of oxidative stress. ER stress induced by tunicamycin caused an increase in oxidative stress in both the kidneys of old and young mice, but the increase was more prominent in those of old mice. When old mice were pretreated with antioxidant NAC or BHA before tunicamycin challenge, renal injury was largely prevented. Moreover, the degree of renal protection was closely related to the extent of oxidative stress inhibition. BHA treatment, which resulted in a more extensive decrease in oxidative stress compared to NAC treatment in this experiment, offered a better renal protection than NAC treatment. It is unclear if different mechanisms of antioxidant actions of NAC and BHA, with NAC serving as a cysteine precursor that increases reduced glutathione [49] and BHA acting against lipid oxidation [50], contribute to the different degrees of protection conferred. Overall, these data suggest that excessive oxidative stress initiated by acute ER stress may cause renal damage.

Since the protection against ER stress-induced renal injury in old mice by antioxidants was associated with improved UPR dynamics, including the recovery of XBP-1 splicing and of the PERK-eIF2*α* pathway, excessive oxidative stress may be one of the reasons for aging kidney failing in UPR. This is partly supported by our finding that high levels of H_2_O_2_ decreased IRE1 levels and XBP-1 splicing, which were largely prevented in the presence of an antioxidant. Future studies are required to determine if oxidants could affect the kinetics of UPR especially PERK phosphorylation and if specific phosphatases are involved in dephosphorylation of PERK in aging kidney under a late time point of acute ER stress. However, since old mice receiving low dose of tunicamycin had the UPR in XBP-1 and phosphorylated PERK similar to those of young mice, it is likely that a different magnitude of oxidative stress may dictate UPR kinetics.

Interestingly, our experiments using young AGER1 transgenic mice showed that a reduction of oxidative stress was sufficient to protect against ER stress in an aging-independent manner. AGER1 is a receptor of AGEs and AGER1 can decrease prooxidant action [51]. Previous reports have demonstrated that the levels of AGEs were increased in cases of acute kidney injury caused by various reasons including ischemia or endotoxin [45, 51, 52]. Since inhibition of AGEs by aminoguanidine has been shown to decrease acute kidney injury caused by various diseases [53, 54], these data, together with our findings from AGER1 transgenic mice, further support the proposition that AGEs are an important source of oxidative stress that is directly involved in acute kidney injury.

Drug nephrotoxicity is increased in elderly patients due partly to the decreased capability to metabolize and excrete drugs [55]. Theoretically, this may also be the case for tunicamycin and thus lead to increased ER stress kidney injury in old mice. However, we did not find tunicamycin accumulation in old mice in the dosage used in this study. Moreover, we found that the same dose of tunicamycin caused more cell death directly in proximal tubules isolated from old mice, suggesting that aging kidney is intrinsically more susceptible to severe ER stress-induced renal injury even though we still need to rule out a possibility that tunicamycin acts more strongly to inhibit protein N-glycosylation in the kidneys of old mice.

## Data Availability

The data used to support the findings of this study are available from the corresponding author upon request.

## Supplementary Materials

Supplementary Materials Supplementary Figure 1: severe ER stress-induced kidney injury in old mice. Both old and young mice were injected with 0.2 *μ*g/g of tunicamycin. Renal histology was examined 72 hours after injection (*n* = 6/age group). While renal tubules remained relative intact in young mice ((a) 200x, PAS), the formation of a big vacuole in proximal tubules was prominent in old mice ((b) 200x, PAS), which affected 61% of proximal tubules in the cortex (c). TUNEL staining showed more apoptotic cells (arrows) in the kidneys of old mice ((d) representative section of young mice; (e) representative section of old mice, 400x). (f) The number of apoptotic cells per high-power field was more in old mice. Scale bar = 50 *μ*m. ^∗∗^ *p* < 0.01 vs. young mice. Supplementary Figure 2: no differences in blood tunicamycin levels between old and young mice. Old and young mice were injected with 0.8 *μ*g/g of tunicamycin, and blood was obtained from mice 0.5, 1, and 2 hours after injection. Plasma tunicamycin levels were determined by HPLC. Peak drug levels were observed in both old and young mice at 1 hour after injection and were comparable between young and old mice. Supplementary Figure 3: tunicamycin induced more cell death in proximal tubules isolated from old mice: proximal tubules isolated from old and young mice were exposed to increasing concentration of tunicamycin (0.5–5 *μ*g/ml) for 24 hours. Cell death was determined by LDH release from the cells, and the data was expressed as the ratio of LDH in medium to total LDH from both cells and medium. ^∗^ *p* < 0.05 vs. young proximal tubules treated with the same dose of tunicamycin. Supplementary Figure 4: electron microscopic examination of renal lesions of old mice with ER stress injury: extensive vacuolation was present in old, but not in young, proximal tubular cells of mice ((a) young; (b) old). Scale bar = 2.0 *μ*m. Higher magnification further revealed the abnormalities in mitochondria and rough ER in old mice (scale bar = 500 nm). Mitochondria contained condensed body and lost the regular structure of cristae ((c) arrow). (d) Many round-shaped dilated ER with ribosomes still attached to the outside membrane were seen (red arrow) and may appear as a vacuole under a light microscope. The yellow arrow points to a membrane-bounded, multilayered inclusion body and an inclusion body containing incompletely digested organelles. Supplementary Figure 5: differences in mRNA expression of UPR-related genes in the kidneys of old and young mice at baseline. Renal cortex RNA was obtained from normal old and young mice (*n* = 4/age group). mRNA levels of GRP78, GRP94, OPR-150, IRE1, XBP-1, and CHOP were measured by real-time PCR and corrected for *β*-actin mRNA levels. The levels in the kidneys from young mice were arbitrarily defined as 1. ^∗^ *p* < 0.05 and ^∗∗^ *p* < 0.01 vs. the levels in the kidneys from young mice. Supplementary Figure 6: GRP78 and GRP94 immunohistochemistry: renal sections from normal young mice (*n* = 3) were stained with anti-GRP78 or anti-GRP94, and the positive staining was revealed by FITC. To visualize the segment of tubules positive for GRP78 and GRP94, AQP1 that marks proximal tubules and THP that marks thick ascending limbs and distal convoluted tubules were stained and labeled (Cy5). Additionally, cell nuclei were stained with blue DAPI. (a) and (b) panels clearly showed that the relatively strong GRP78 and GRP94 staining colocalized with THP-positive tubules. (c) and (d) panels indicated that neither GRP78 nor GRP94 strong staining was present in AQP1-positive tubules. Scale bar = 25 *μ*m. Supplementary Figure 7: differences in expression of UPR-related genes between the kidneys of old and young mice after low dose of tunicamycin injury: renal cortex RNA was obtained from young and old mice at baseline and 72 hours after low dose (0.2 *μ*g/g) of tunicamycin injection. The levels of GRP78, GRP94, ORP150, EDEM1, and CHOP mRNAs were measured by real-time PCR, and the results were corrected by *β*-actin mRNA levels. The presence of spliced XBP-1 was visualized by regular PCR. GRP78, GRP94, CHOP, and caspase 12 protein levels were determined by Western blots. *β*-actin levels were measured at the same membrane. The intensity of Western blot band was quantified using a densitometer. (a) mRNA levels at baseline and 72 hours after tunicamycin injection. ^∗∗^ *p* < 0.01 vs. mRNA levels in young mice at 72 hours. XBP-1 splicing, which was not seen in young control (Y/c) and old control (O/c) mice, was clearly present in tunicamycin-treated young mice (Y/tuni) and old mice (O/tuni). (b) GRP78 and GRP94 protein levels were determined (8 mice/age/time point). Representative gels from two kidneys of young and old mice at baseline and 72 hours after tunicamycin injection. Y: young mice control; O: old mice control; YT: young mice with 0.2 *μ*g/gBW tunicamycin; OT: old mice with 0.2 *μ*g/gBW tunicamycin. Density of the specific band was quantitated. ^∗^ *p* < 0.05 and ^∗∗^ *p* < 0.01 vs. protein levels in young mice at 72 hours. (c) CHOP and caspase 12 protein levels at 72 hours after tunicamycin injection. Two representative gels from the kidneys of old and young mice showed that CHOP protein levels were higher in old mice and cleaved caspase 12 was only present in old mice. Supplementary Figure 8: oxidative stress and IRE1-XBP-1. (a) Severe oxidative stress decreased IRE1 mRNA levels. RNA was collected from proximal tubular cells treated with 1 and 3 mM of H_2_O_2_ in the presence or absence of NAC. mRNA levels of IRE1 were determined by real-time PCR and corrected for *β*-actin mRNA levels. The levels in cells without receiving H_2_O_2_ were arbitrarily defined as 1. ^∗∗^ *p* < 0.01 vs. cells without receiving H_2_O_2_ (0). ^##^ *p* < 0.01 vs. cells treated with 1 mM of H_2_O_2_. (b) Severe oxidative stress decreased the levels of spliced XBP-1 in proximal tubular cells. Spliced XBP-1 was readily present in cultured proximal tubular cells. Adding high dose of H_2_O_2_ (1–3 mM) into these cells for 6 hours caused a decrease in spliced XBP-1 mRNA levels. Pretreatment of cells with 15 mM of NAC 1 hour before adding H_2_O_2_ blocked the effect of H_2_O_2_. (c) Severe oxidative stress decreased protein levels of spliced XBP-1 and IRE1. Proximal tubular cells were treated with different concentrations of H_2_O_2_ (0.5–3 mM) for 24 hours, in the presence or absence of NAC pretreatment. Nuclear protein was collected for the measurement of spliced XBP-1, and protein from total cell lysate was collected for the determination of IRE1. The blots used for IRE1 Western blot were reprobed with *β*-actin. High concentration of H_2_O_2_ decreases the levels of both spliced XBP-1 and IRE1. The presence of NAC blocked the effect of H_2_O_2_. Supplementary Figure 9: protection against ER stress renal injury by overexpressing AGER1. AGER1 transgenic and wild-type mice were treated with high dose of tunicamycin. Severe renal injury characterized by extensive vacuolation and tubular cell death was present in wild-type mice (a) while the injury was much less in AGER1 transgenic mice (b). (c) Morphometry analysis revealed that tubular damage occurred in 62% of proximal tubules in wild-type mice while the injured area was reduced by 50% in the kidneys of transgenic mice. ^∗^ *p* < 0.05 vs. AGER1 transgenic mice. Data was expressed as mean ± SD.
