# Supplementary material for: Excessive Oxidative Stress Contributes to Increased Acute ER Stress Kidney Injury in Aged Mice
Source: Oxid Med Cell Longev. 2019 Jan 28;2019:2746521. doi: 10.1155/2019/2746521 (PMC6369482; doi:10.1155/2019/2746521)
Supplement: Supplementary Materials — Supplementary Figure 1: severe ER stress-induced kidney injury in old mice. Both old and young mice were injected with 0.2 μg/g of tunicamycin. Renal histology was examined 72 hours after injection (n = 6/age group). While renal tubules remained relative intact in young mice ((a) 200x, PAS), the formation of a big vacuole in proximal tubules was prominent in old mice ((b) 200x, PAS), which affected 61% of proximal tubules in the cortex (c). TUNEL staining showed more apoptotic cells (arrows) in the kidneys of old mice ((d) representative section of young mice; (e) representative section of old mice, 400x). (f) The number of apoptotic cells per high-power field was more in old mice. Scale bar = 50 μm. ∗∗ p < 0.01 vs. young mice. Supplementary Figure 2: no differences in blood tunicamycin levels between old and young mice. Old and young mice were injected with 0.8 μg/g of tunicamycin, and blood was obtained from mice 0.5, 1, and 2 hours after injection. Plasma tunicamycin levels were determined by HPLC. Peak drug levels were observed in both old and young mice at 1 hour after injection and were comparable between young and old mice. Supplementary Figure 3: tunicamycin induced more cell death in proximal tubules isolated from old mice: proximal tubules isolated from old and young mice were exposed to increasing concentration of tunicamycin (0.5–5 μg/ml) for 24 hours. Cell death was determined by LDH release from the cells, and the data was expressed as the ratio of LDH in medium to total LDH from both cells and medium. ∗ p < 0.05 vs. young proximal tubules treated with the same dose of tunicamycin. Supplementary Figure 4: electron microscopic examination of renal lesions of old mice with ER stress injury: extensive vacuolation was present in old, but not in young, proximal tubular cells of mice ((a) young; (b) old). Scale bar = 2.0 μm. Higher magnification further revealed the abnormalities in mitochondria and rough ER in old mice (scale bar = 500 nm). Mitochondria con [file 2746521.f1.pdf]

Supplementary Materials:

Supplementary Figure 1

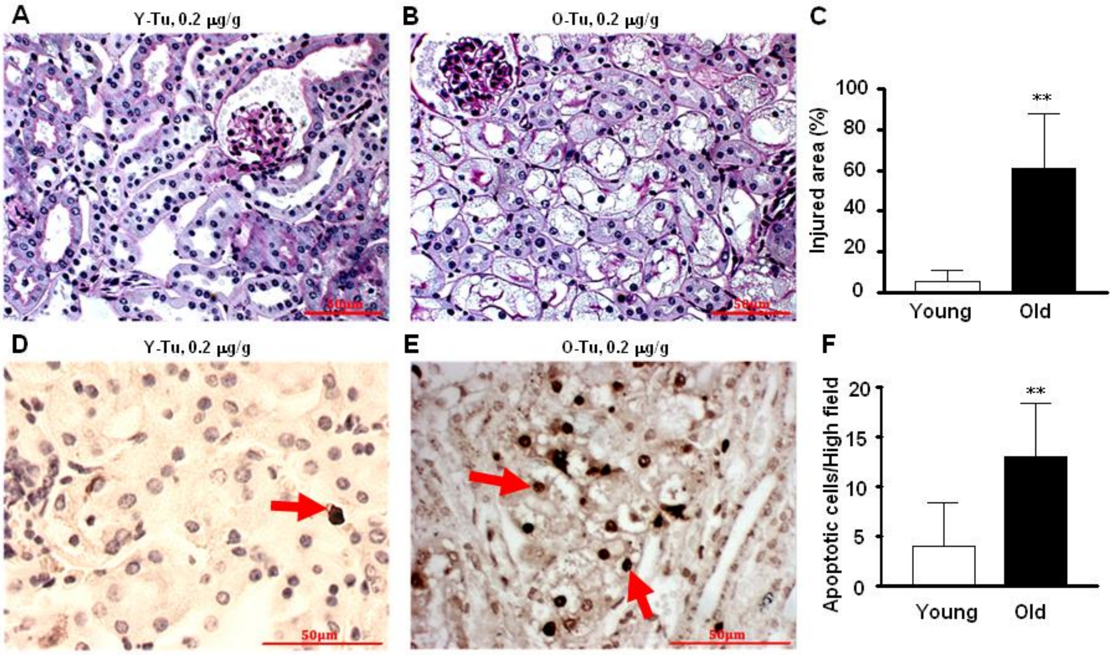

Supplementary Figure 1. Severe ER stress induced kidney injury in old mice. Both old and young mice were injected with 0.2 μg/g of tunicamycin. Renal histology was examined 72 hours after injection (n=6/age group). While renal tubules remained relative intact in young mice (**A**, 200×, PAS), the formation of big vacuole in proximal tubules was prominent in old mice (**B,** 200×, PAS), which affected 61% of proximal tubules in cortex (**C**). TUNEL staining showed more apoptotic cells (arrows) in the kidneys of old mice (**D,** representative section of young mice; **E**, representative section of old mice, 400×). (**F**) The number of apoptotic cells per high power field was more in old mice. Scale bar=50 μm. **p<0.01, *vs*. young mice.

Supplementary Figure 2

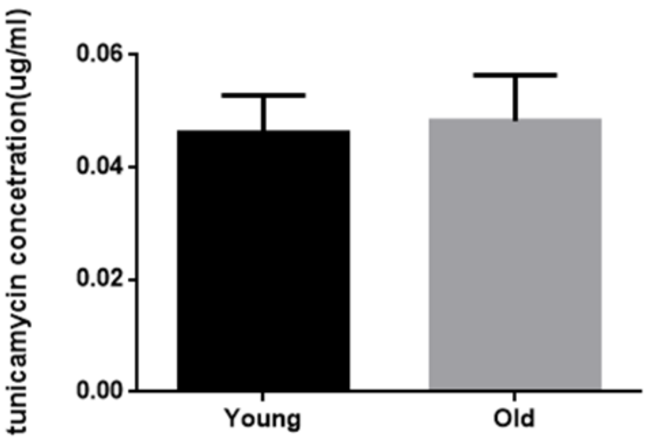

Supplementary Figure 2. No differences in blood tunicamycin levels between old and young mice. Old and young mice were injected with 0.8 μg/g of tunicamycin and blood was obtained from mice 0.5, 1, and 2 hours after injection. Plasma tunicamycin levels were determined by HPLC. Peak drug levels were observed in both old and young mice at 1 hour after injection and were comparable between young and old mice.

Supplementary Figure 3

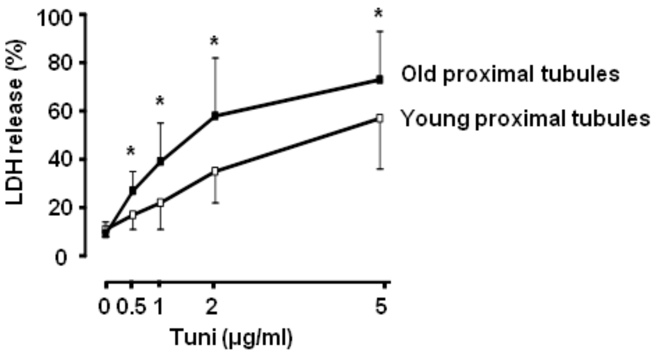

Supplementary Figure 3. Tunicamycin induced more cell death in proximal tubules isolated from old mice: Proximal tubules isolated from old and young mice were exposed to increasing concentration of tunicamycin (0.5-5 μg/ml) for 24 hours. Cell

death was determined by LDH release from the cells and the data was expressed as the ratio of LDH in medium to total LDH from both cells and medium. $*p<0.05$, *vs*. young proximal tubules treated with the same dose of tunicamycin.

Supplementary Figure 4

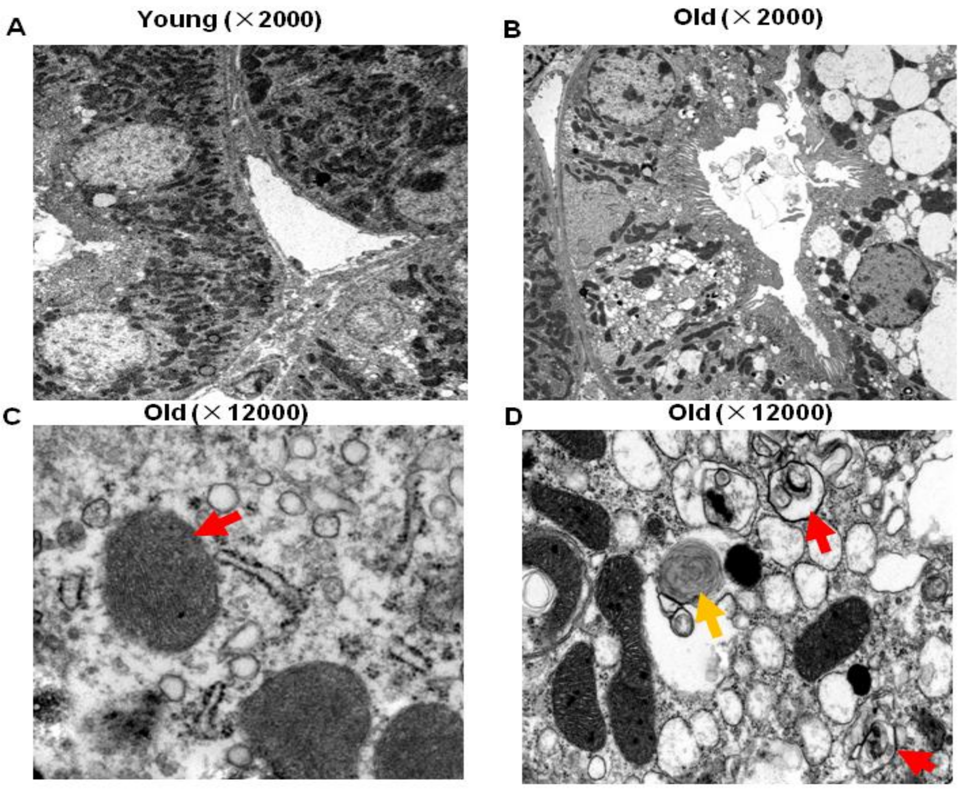

Supplementary Figure 4. Electron microscopic examination of renal lesions of old mice with ER stress injury: Extensive vacuolation was present in old, but not in young mice proximal tubular cells (**A,** young; **B,** old). Scale bar=2.0 μm. Higher magnification further revealed the abnormalities in mitochondria and rough ER in old mice (Scale bar=500 nm). Mitochondria contained condensed body and lost the regular structure of cristae (**C**, arrow). (**D**) Many round-shape dilated ER with ribosomes still attached to outside membrane were seen (red arrow) and may appear as vacuole under light

microscope. Yellow arrow points to a membrane-bounded, multilayered inclusion body and an inclusion body containing incompletely digested organelles.

Supplementary Figure 5

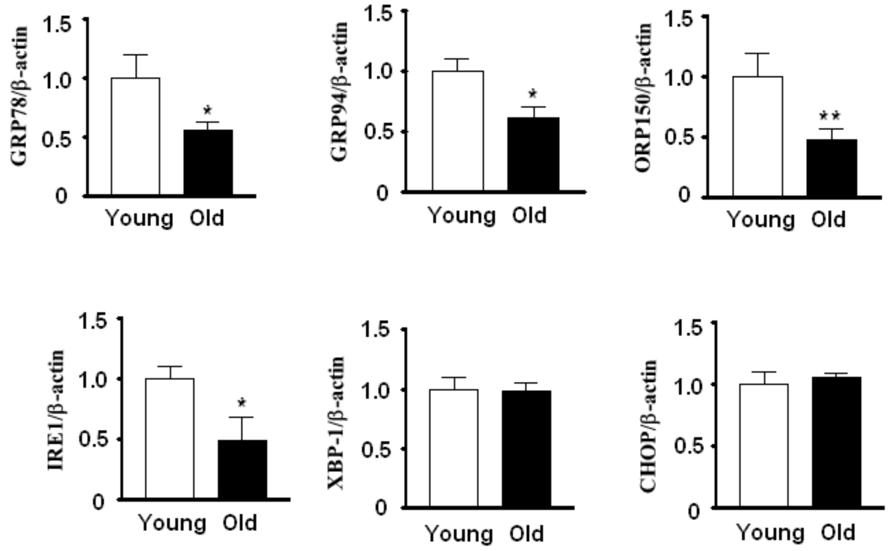

Supplementary Figure 5. Differences in mRNA expression of UPR related genes in kidneys of old and young mice at baseline. Renal cortex was obtained from normal old and young mice (n=4/age group). mRNA levels of GRP78, GRP94, OPR-150, IRE1, XBP-1, and CHOP were measured by real time PCR and corrected for β-actin mRNA levels. The levels in kidneys from young mice were arbitrarily defined as 1. *p<0.05, **p<0.01, *vs*. the levels in kidneys from young mice.

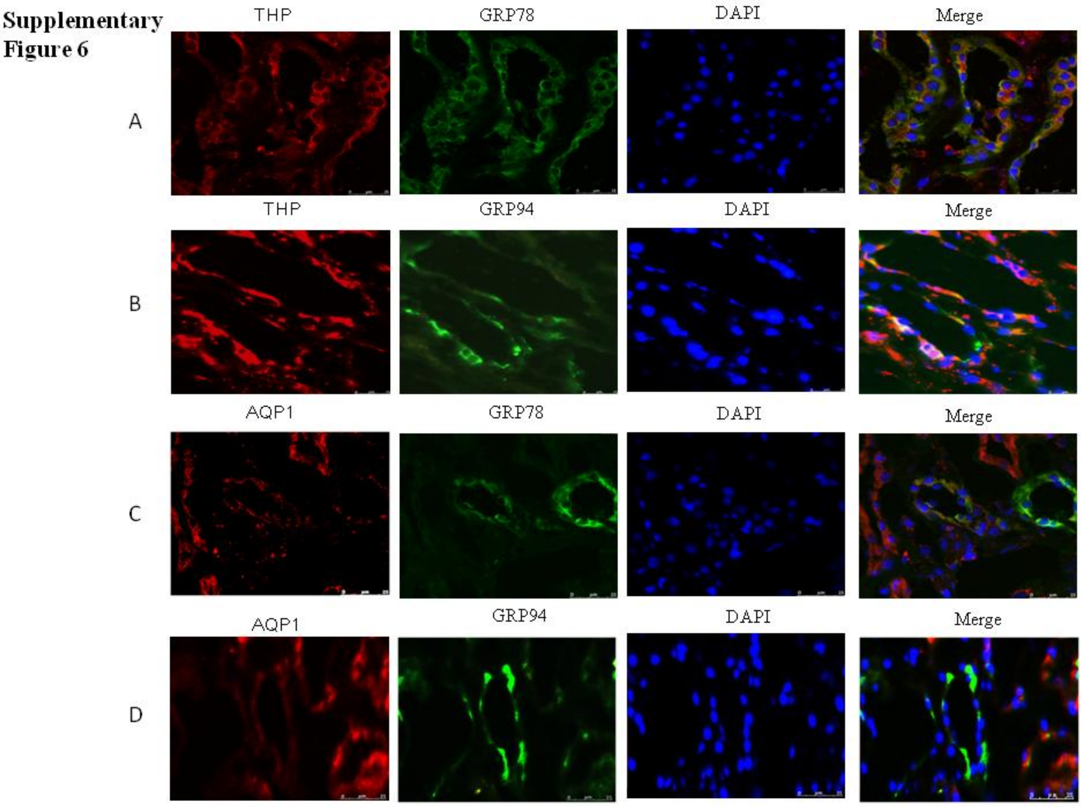

Supplementary Figure 6. GRP78 and GRP94 immunohistochemisty: Renal sections from normal young mice (n=3) were stained with anti-GRP78 or anti-GRP94 and the positive staining were revealed by FITC. To visualize the segment of tubules positive for GRP78 and GRP94, AQP1 that marks proximal tubules and THP that marks thick ascending limbs and distal convoluted tubules were stained and labeled (Cy5). Additionally, cell nuclei were stained with blue DAPI. (**A**) and (**B**) panels clearly showed that the relatively strong GRP78 and GRP94 staining co-localized with THP positive tubules. (**C**) and (**D**) panels indicated that neither GRP78 nor GRP94 strong staining were present in AQP1 positive tubules. Scale bar = 25μ m.

Supplementary Figure 7

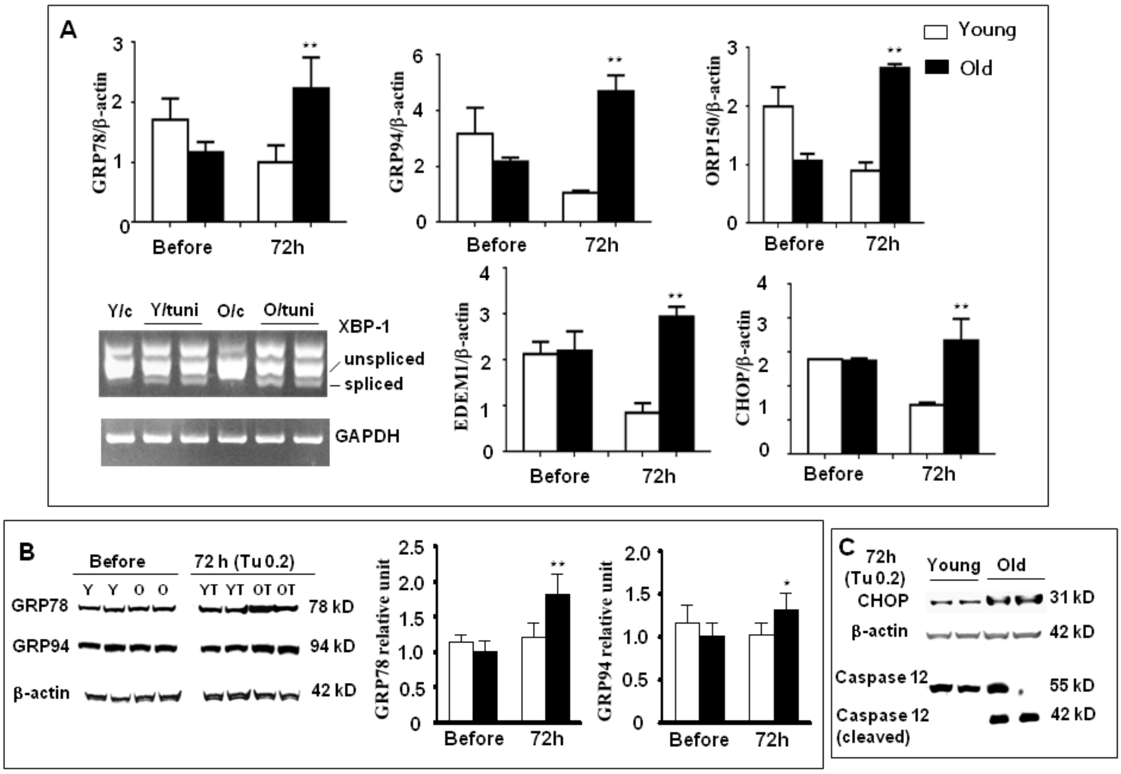

Supplementary Figure 7. Differences in expression of UPR related genes between old and young mice kidneys after low dose of tunicamycin injury: Renal cortex RNA was obtained from young and old mice at baseline and 72 hours after low dose (0.2 μg/g) of tunicamycin injection. The levels of GRP78, GRP94, ORP150, EDEM1, and CHOP mRNA were measured by real-time PCR and the results were corrected by β–actin mRNA levels. The presence of spliced XBP-1 was visualized by regular PCR. GRP78, GRP94, CHOP, and caspase 12 protein levels were determined by western blots. β–actin levels were measured at the same membrane. The intensity of western blot band was quantified using a densitometer. (**A**) mRNA levels at baseline and 72 hours after tunicamycin injection. **p<0.01, *vs.* mRNA levels in young mice at 72 hours. XBP-1 splicing, which was not seen in young control (Y/c) and old control (O/c), was clearly

present in tunicamycin treated young mice (Y/tuni) and old mice (O/tuni). (**B**) GRP78 and GRP94 protein levels were determined (8 mice/age/time point). Representative gels from two kidneys of young and old mice at baseline and 72 hours after tunicamycin injection. Y=Young mice control; O=Old mice control; YT=Young mice with 0.2μg/gBW Tunicamycin; OT=Old mice with 0.2μg/gBW Tunicamycin. Density of specific band was quantitated. *p<0.05, **p<0.01, *vs.* protein levels in young mice at 72 hours. (**C**) CHOP and caspase 12 protein levels at 72 hours after tunicamycin injection. Two representative gels from old and young mice kidneys showed that CHOP protein levels were higher in the old and cleaved caspase 12 was only present in the old.

Supplementary Figure 8

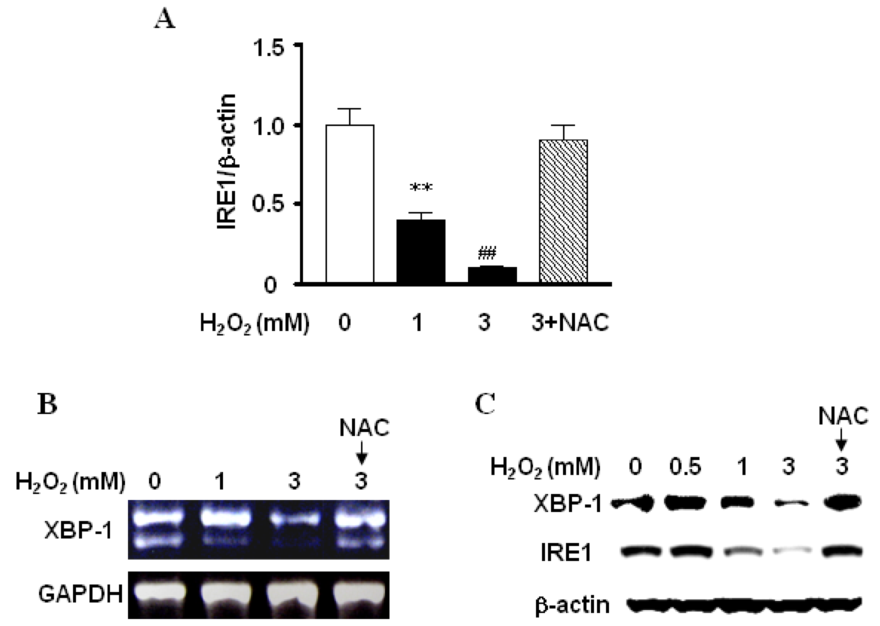

Supplementary Figure 8. Oxidative stress and IRE1-XBP-1. (**A**) Severe oxidative stress decreased IRE1 mRNA levels. RNA was collected from proximal tubular cells treated with 1 and 3 mM of H_2O_2 in the presence or absence of NAC. mRNA levels of

IRE1 were determined by real-time PCR and corrected for β-actin mRNA levels. The levels in cells without receiving H_2O_2 were arbitrarily defined as 1. **p<0.01, *vs*. cells without receiving H_2O_2 (0). ##p<0.01, *vs*. cells treated with 1 mM of H_2O_2. (**B**) Severe oxidative stress decreased the levels of spliced XBP-1 in proximal tubular cells. Spliced XBP-1 was readily present in cultured proximal tubular cells. Adding high dose of H_2O_2 (1-3 mM) into these cells for 6 hours caused a decrease in spliced XBP-1 mRNA levels. Pretreatment of cells with 15 mM of NAC 1 hour before adding H_2O_2 blocked the effect of H_2O_2. (**C**) Severe oxidative stress decreased protein levels of spliced XBP-1 and IRE1. Proximal tubular cells were treated with different concentration of H_2O_2 (0.5-3 mM) for 24 hours, in the presence or absence of NAC pretreatment. Nuclear protein was collected for the measurement of spliced XBP-1 and protein from total cell lysate was collected for the determination of IRE1. The blots used for IRE1 western blot were re-probed with β-actin. High concentration of H_2O_2 decreases the levels of both spliced XBP-1 and IRE1. The presence of NAC blocked the effect of H_2O_2.

Supplementary Figure 9

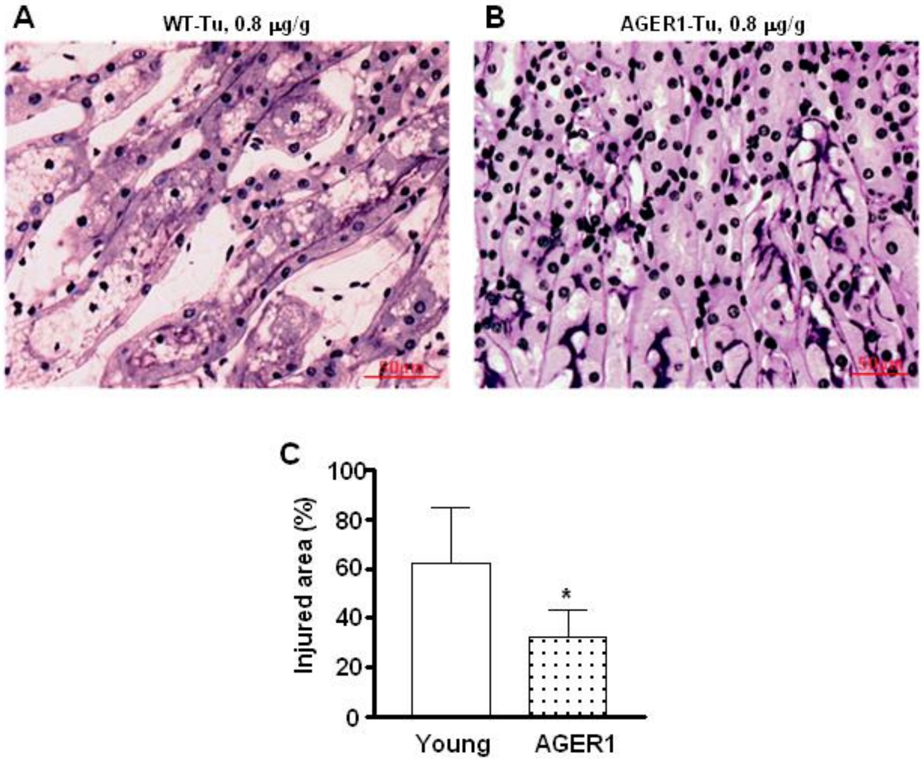

Supplementary Figure 9. Protection against ER stress renal injury by overexpressing AGER1. AGER1 transgenic and wild type mice were treated with high dose of tunicamycin. Severe renal injury characterized by extensive vacuolation and tubular cell death were present in wild type mice (**A**) while the injury was much less in AGER1 transgenic mice (**B**). (**C**) Morphometry analysis revealed that tubular damage occurred 62% of proximal tubules in wild type mice while the injured area was reduced 50% in kidneys of transgenic mice. *$p<0.05$, *vs*. AGER1 transgenic mice. Data was expressed as mean±SD.
